# Massive suprachoroidal hemorrhage in dengue-associated HLH: A clinical image

**DOI:** 10.1016/j.idcr.2025.e02371

**Published:** 2025-09-13

**Authors:** Jessel Dsouza, May D'souza

**Affiliations:** Department of Ophthalmology, Father Muller Medical College Hospital, Mangalore, India

**Keywords:** Suprachoroidal hemorrhage, Dengue fever, Hemophagocytic lymphohistiocytosis (HLH) Ocular emergency, Multidisciplinary care, Vision-threatening complication, Terson syndrome

## Abstract

Dengue fever can manifest with severe hemorrhagic complications, though ocular involvement is rare. We report a unique case of a 44-year-old male with dengue hemorrhagic fever complicated by secondary hemophagocytic lymphohistiocytosis (HLH) and severe thrombocytopenia, who developed a massive suprachoroidal hemorrhage culminating in spontaneous auto-evisceration and requiring surgical evisceration. To our knowledge, this is the first reported case of dengue-associated HLH presenting with such a catastrophic ocular complication. This case underscores the importance of early ophthalmic evaluation and multidisciplinary intervention in systemic viral illnesses.

## Introduction

Dengue hemorrhagic fever (DHF) is a severe form of dengue virus infection, characterized by thrombocytopenia, capillary leakage, and bleeding manifestations. Ocular complications,although uncommon,can include retinal hemorrhages, maculopathy, and suprachoroidal hemorrhage [1]. Hemophagocytic lymphohistiocytosis (HLH), a rare hyperinflammatory syndrome, can further exacerbate hemorrhagic complications [2]. This report highlights an unusual case of DHF with HLH, complicated by intracerebral hemorrhage and massive suprachoroidal hemorrhage, which progressed to auto-evisceration and required surgical evisceration of the affected eye.

## Case presentation

A 44-year-old male with no known comorbidities presented with a five-day history of high-grade fever (maximum 102 °F), generalized weakness and headache. On admission he was febrile but hemodynamically stable. Initial investigations showed severe thrombocytopenia (platelet count 14,000/µL) and raised inflammatory markers. Dengue NS1 antigen was positive.

Laboratory evaluation revealed profound thrombocytopenia (nadir 7000/µL on Day 3), markedly elevated serum ferritin (>10,000 ng/mL), raised lactate dehydrogenase (862 IU/L) and elevated C-reactive protein (25.71 mg/L). Liver function tests were deranged.

The treating team made a clinical diagnosis of secondary HLH because the patient fulfilled several HLH-2004 elements (persistent high fever, extreme hyperferritinemia >10,000 ng/mL, and severe cytopenia). In the context of urgent clinical deterioration, not all confirmatory tests recommended by HLH-2004 (bone-marrow biopsy, serum triglycerides/fibrinogen, soluble CD25, NK-cell testing) were obtained during the acute admission; hence the diagnosis was based on the overall clinical and laboratory picture.

He was admitted under General Medicine with dengue hemorrhagic fever complicated by HLH and severe thrombocytopenia. In the SICU, he received intravenous corticosteroids, supportive care and multiple platelet transfusions. Serial platelet counts and transfusions included:

Day 1 (admission): Platelet 14,000/µL

Day 2: Platelet transfusion (2 pints)

Day 3: Platelet nadir 7000/µL; platelet transfusion (4 pints); MRI brain performed

Day 10: Platelet 188,000/µL

Day 13 (discharge): Platelet 255,000/µL; ferritin 1830 ng/mL; CRP 6.84 mg/L

On Day 3, his sensorium worsened, oxygen saturation dropped and tachycardia developed, prompting ICU transfer. Neurology consultation and MRI brain revealed multiple petechial hemorrhages in the basal ganglia with edema and a small right-eye vitreous hemorrhage (Terson syndrome).

**Fig. 1:**
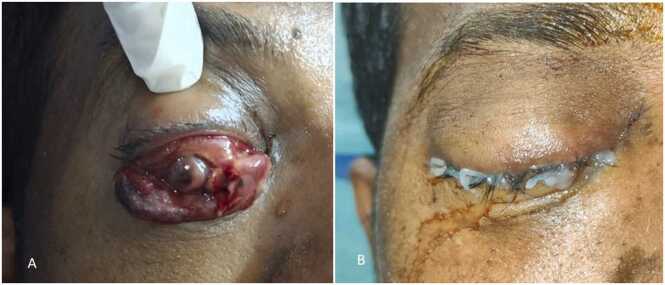
(Fig. 1A) External photograph showing auto-evisceration of the right globe in a 44-year-old male with dengue-associated HLH, following massive suprachoroidal hemorrhage. (Fig. 1B) Post-operative photograph after surgical evisceration, showing conjunctival wound closure with sutures in place.

Ocular involvement: On Day 5, ophthalmic examination showed conjunctival chemosis, corneal haziness, proptosis and a sluggish pupil in the right eye. Despite topical therapy and tight dressings, the right eye worsened. By Day 7, there was severe conjunctival necrosis, a non-reactive pupil and no light perception; a massive suprachoroidal hemorrhage was diagnosed clinically. External photograph demonstrates spontaneous auto-evisceration with prolapse of intraocular contents following suprachoroidal hemorrhage (Fig. 1 A).

Surgical intervention: On Day 9, right-eye evisceration was performed under general anaesthesia. Intra-operative findings included hemorrhagic choroid with necrotic intraocular contents and no viable retina. The sclera and conjunctiva were reconstructed with closure (Fig. 1B). Histopathology confirmed hemorrhagic choroid without residual retina.

Microbiology: Blood and ocular cultures were negative for bacterial or fungal growth.

Outcome: The patient remained hemodynamically stable postoperatively. Platelets and inflammatory markers normalized, neurological status improved, and he was discharged on Day 13. At discharge, left eye visual acuity was 6/6 with a normal anterior segment. He was counselled regarding permanent right-eye vision loss and advised regular ophthalmology follow-up.

## Discussion

Dengue infection is increasingly recognized to involve the eye with a wide spectrum of findings. Retinal hemorrhages, vasculitis, optic neuritis, and maculopathy have all been reported in dengue patients¹ . A recent case series noted ocular complications in roughly 10 % of dengue cases, often presenting 1–2 weeks after fever onset¹ . Hemorrhagic findings (e.g., retinal hemorrhages) are common, but massive intraocular bleeding is exceedingly rare. Notably, severe thrombocytopenia in dengue can precipitate a retrobulbar hemorrhage accompanied by vitreous and even suprachoroidal hemorrhage, leading to globe perforation³ . Such catastrophic bleeding, reminiscent of Terson-like syndromes, underscores that dengue can rarely cause vision-threatening intraocular hemorrhage.

Hemophagocytic lymphohistiocytosis (HLH) is a life-threatening hyperinflammatory syndrome marked by uncontrolled macrophage and T-cell activation². It can be primary (genetic) or secondary to triggers such as infection. Dengue virus has emerged as a significant infectious trigger of secondary HLH². Dengue-associated HLH typically produces persistent high fevers, hepatosplenomegaly, hyperferritinemia, and severe pancytopenia (including profound thrombocytopenia)². These features strongly overlap with severe dengue, making the diagnosis challenging. In our patient, HLH compounded the coagulopathy of dengue; the extreme cytopenias and “cytokine storm” of HLH likely amplified the bleeding risk.

Suprachoroidal hemorrhage (SCH) is classically an uncommon but devastating complication of intraocular surgery or blunt trauma [4]. In SCH, blood accumulates between the choroid and sclera, forcing the retina inward; if unchecked, it can extrude intraocular contents. Spontaneous SCH without prior ocular injury is exceedingly rare and almost always occurs in the presence of systemic risk factors⁴. Known predisposing factors include advanced age, hypertension, valsalva or coughing episodes, anticoagulant/antiplatelet use, and most importantly thrombocytopenia⁴. For example, Cruz-Pimentel et al. reported spontaneous SCH in a patient with chronic leukemia and severe thrombocytopenia [3], [4]. In our dengue-HLH patient, similarly profound thrombocytopenia and vascular fragility likely precipitated the massive SCH. The unique feature here is auto-evisceration of the globe: as the hemorrhage progressed, intraocular pressure rose until the eye literally began to expel its contents. This sequence resembles “expulsive hemorrhage” classically seen in endophthalmitis or intraoperative SCH, but to our knowledge it has never been reported in dengue or HLH.

To our knowledge, this case is unprecedented. We found no prior reports of spontaneous SCH leading to globe auto-evisceration in any dengue patient, nor any HLH-related ocular case similar to this. Its rarity underscores its clinical significance. This case expands the spectrum of dengue-associated ocular complications and illustrates how HLH can dramatically worsen hemorrhagic outcomes. Clinically, it reinforces the importance of early ophthalmologic evaluation in dengue patients with severe systemic bleeding or visual symptoms. It also highlights that in dengue complicated by HLH, clinicians should aggressively correct coagulopathy and monitor for intraocular hemorrhage. In these respects, our findings are consistent with recent literature emphasizing multidisciplinary care in dengue and in HLH [1], [2].

In summary, we report an exceptional case of massive suprachoroidal hemorrhage with globe auto-evisceration in dengue fever complicated by secondary HLH. By situating our case within recent studies of dengue’s ocular disease and HLH[1], [2], [4]we demonstrate a novel and devastating complication of these syndromes. This report underscores the need for heightened vigilance and prompt ophthalmic assessment in severe dengue and HLH, and adds to the limited literature on vision-threatening sequelae in systemic infection.

## Conclusion

Severe dengue complicated by secondary HLH can lead to catastrophic ocular hemorrhages. Vigilant ophthalmic monitoring, early recognition of HLH, and prompt surgical management are key to optimizing systemic and ocular outcomes.

## Ethical approval

Not applicable for single case reports as per institutional and journal policy.

## Role of the funding source

No funding was received for this work. The authors were not paid by any pharmaceutical company or agency to write this article. The authors had full access to all the data in the study and accept responsibility for the decision to submit for publication.

## Consent

Written informed consent for publication of the clinical details and images was obtained from the patient and is available on request.

## Consent

Written informed consent was obtained from the patient for publication of this case report and accompanying images. A copy of the written consent is available for review by the Editor-in-Chief of this journal on request.

## Funding

No funding was received for the preparation of this manuscript.

## CRediT authorship contribution statement

**Jessel Dsouza:** Writing – review & editing, Writing – original draft, Visualization, Project administration, Methodology, Investigation, Formal analysis, Data curation, Conceptualization. **May D'souza:** Writing – review & editing, Validation, Supervision.

## Declaration of Competing Interest

The authors declare no financial or personal relationships with other people or organizations that could inappropriately influence or bias their work. They have nothing to declare.
